# Community participation for transformative action on women’s, children’s and adolescents’ health

**DOI:** 10.2471/BLT.15.168492

**Published:** 2016-05-02

**Authors:** Cicely Marston, Rachael Hinton, Stuart Kean, Sushil Baral, Arti Ahuja, Anthony Costello, Anayda Portela

**Affiliations:** a London School of Hygiene & Tropical Medicine, London, England.; b Partnership for Maternal, Newborn & Child Health, Geneva, Switzerland.; c World Vision International, Milton Keynes, England.; d Health Research and Social Development Forum (HERD), Kathmandu, Nepal.; e Department of Health and Family Welfare, Government of Odisha, Bhubaneswar, India.; f World Health Organization, Avenue Appia 20, 1211 Geneva 27, Switzerland.

## Abstract

The *Global strategy for women’s, children’s and adolescents’ health (2016–2030)* recognizes that people have a central role in improving their own health. We propose that community participation, particularly communities working together with health services (co-production in health care), will be central for achieving the objectives of the global strategy. Community participation specifically addresses the third of the key objectives: to transform societies so that women, children and adolescents can realize their rights to the highest attainable standards of health and well-being. In this paper, we examine what this implies in practice. We discuss three interdependent areas for action towards greater participation of the public in health: improving capabilities for individual and group participation; developing and sustaining people-centred health services; and social accountability. We outline challenges for implementation, and provide policy-makers, programme managers and practitioners with illustrative examples of the types of participatory approaches needed in each area to help achieve the health and development goals.

## Résumé

La Stratégie mondiale pour la santé de la femme, de l'enfant et de l'adolescent (2016-2030) reconnaît que les individus jouent un rôle central dans l'amélioration de leur propre santé. Nous pensons que la participation communautaire, et notamment des communautés qui travaillent avec les services de santé (coproduction en matière de soins de santé), sera essentielle pour atteindre les objectifs de la stratégie mondiale. La participation communautaire répond plus particulièrement au troisième objectif clé: transformer les sociétés afin que les femmes, les enfants et les adolescents puissent exercer leur droit de jouir du meilleur état de santé et de bien-être possible. Dans le présent rapport, nous examinons ce que ceci suppose dans la pratique. Nous analysons trois domaines interdépendants dans le cadre d'une action visant une plus grande participation du public en matière de santé: améliorer la capacité de participation des individus et des groupes, développer et assurer la pérennité des services de santé axés sur l'être humain, et favoriser la responsabilisation sociale. Nous donnons par ailleurs un aperçu des difficultés liées à la mise en œuvre et fournissons aux décideurs, aux administrateurs de programmes et aux professionnels de la santé des exemples illustrant les différents types d'approches participatives nécessaires dans chaque domaine pour atteindre les objectifs liés à la santé et au développement.

## Resumen

La Estrategia Mundial para la Salud de la Mujer, el Niño y el Adolescente (2016-2030) reconoce que las personas tienen un papel fundamental a la hora de mejorar su propia salud. Proponemos que la participación comunitaria, concretamente las comunidades que trabajan junto con los servicios sanitarios (coproducción de atención sanitaria), sea fundamental para lograr los objetivos de la estrategia mundial. En particular, la participación comunitaria aborda el tercer objetivo fundamental: transformar las sociedades de modo que las mujeres, los niños y los adolescentes puedan ejercer sus derechos de salud y bienestar en la mayor medida posible. En este artículo se examina lo que esto implica en la práctica. Se debaten tres ámbitos de acción interdependientes que se encaminan hacia una mayor participación del público en la sanidad: mejorar las capacidades para la participación individual y en grupo; desarrollar y mantener servicios sanitarios centrados en las personas; y contabilidad social. Se repasan los desafíos para su implementación, y se ofrece a los responsables políticos, gestores de programas y médicos ejemplos ilustrativos de los tipos de enfoques de participación necesarios en cada ámbito para contribuir a alcanzar los objetivos sanitarios y de desarrollo.

## ملخص

تقر الاستراتيجية العالمية لصحة النساء والأطفال والمراهقين (من عام 2016 إلى عام 2030) أن الأشخاص لديهم دور محوري في تحسين صحتهم بأنفسهم. ونحن نقترح أن المشاركة المجتمعية، وخاصةً المجتمعات التي تعمل إلى جانب مقدمي الخدمات الصحية (الإنتاج المشترك في مجال الرعاية الصحية)، سوف تكون أساسية لتحقيق أهداف الاستراتيجية العالمية. تتناول المشاركة المجتمعية على وجه التحديد ثلث الأهداف الرئيسية: تحويل المجتمعات حتى يتسنى للنساء والأطفال والمراهقين الحصول على حقوقهم بالتمتع بأعلى المعايير الممكنة من الصحة والرفاهية. ونحن نقوم هنا في هذا التقرير بفحص ما يعنيه ذلك من الناحية العملية. نحن نناقش ثلاثة مجالات مترابطة لاتخاذ إجراءات في سبيل زيادة مشاركة العامة في مجال الصحة: وهي تحسين قدرات المشاركة الفردية والجماعية؛ وتطوير ودعم الخدمات الصحية المرتكزة على الأشخاص؛ والمساءلة الاجتماعية. نحن نضع الخطوط العريضة للتحديات التي تواجه عملية التنفيذ، ونقوم بتزويد صناع القرار ومديري البرامج والعاملين في مجال الصحة بأمثلة توضيحية لأنواع نهج المشاركة اللازمة في كل منطقة للمساعدة في تحقيق أهداف الصحة والتنمية.

## 摘要

《妇女、儿童和青少年健康全球战略（2016-2030 年）》让人们意识到：人们在改善自己的健康方面发挥着主要作用。 我们提议，让社区参与其中，尤其是让社区与公共医疗卫生服务部门共同合作（医疗合作），这将是实现全球战略目标的关键条件。 社区参与具体涉及到第三个主要目标： 去改变社会，从而让妇女、儿童和青少年意识到他（她）们拥有实现最高标准健康和幸福的权利。 在本文中，我们主要研究这在实践中意味着什么。 我们讨论让更多公众参与到健康战略中来所采取举措的三个相互依存的领域： 提高个人和群体的参与能力；发展并保持以人为本的公共医疗卫生服务和社会责任。 我们简略概述了实施时面对的挑战，并为决策者、方案管理者以及从业者提供每个领域所需的参与途径类型的说明例证，以帮助实现健康和发展目标。

## Резюме

В глобальной стратегии охраны здоровья женщин, детей и подростков (2016–2030 гг.) признается центральная роль людей в улучшении своего собственного здоровья. В данной статье предлагается придать участию сообществ, особенно тех, которые сотрудничают со службами здравоохранения (вовлеченных в совместное производство в сфере здравоохранения), главное значение в достижении целей глобальной стратегии. Участие сообществ, в частности, способствует достижению третьей основной цели — преобразовать общества таким образом, чтобы женщины, дети и подростки могли реализовать свои права на наивысший достижимый уровень здоровья и благополучия. В данной статье рассматривается, что это подразумевает на практике. Авторы изучают три взаимосвязанных направления деятельности, нацеленной на увеличение вовлеченности общественности в сферу здравоохранения: увеличение возможностей для участия отдельных лиц и групп, развитие и поддержка служб здравоохранения с учетом человеческого фактора и внедрение механизмов социальной ответственности. Выделяются задачи, которые необходимо решить, а для лиц, формирующих политику, руководителей программ и практикующих врачей приводятся наглядные примеры подходов, предполагающих непосредственное участие, которые необходимо применять в каждом направлении для содействия достижению целей в сфере здравоохранения и развития.

## Introduction

The *Global strategy for women’s, children’s and adolescents’ health (2016–2030)* calls for action towards three objectives for health: survive (end preventable deaths), thrive (ensure health and well-being) and transform (expand enabling environments).^1^ The strategy recognizes that “women, children and adolescents are potentially the most powerful agents for improving their own health and achieving prosperous and sustainable societies”. Global, national and sub-national development policies have until now been largely orientated towards addressing the objectives of helping people to survive and thrive. However, to accomplish the overall objectives of the strategy we need to address the third objective: “to transform societies so that women, children and adolescents everywhere can realize their rights to the highest attainable standards of health and well-being”. Transforming societies requires participation, including communities working together with health services to reach health goals (what is termed co-production). In this paper, we examine what this implies in practice.

Community participation is promoted in global dialogue as a vital element of a human rights-based approach to health. This means not just ensuring the provision of health services and their use by the public but also tackling the underlying social determinants of health.^2^ While proven clinical and health service interventions could save numerous lives by 2030, if they were made available to all, those people most in need of health care are often not reached.^3^^,^^4^ Many factors – wealth, environment, gender, education, geography, culture and other structural determinants – affect health outcomes directly through health services uptake, and indirectly via relationships and behaviours outside the clinic setting.^5^^,^^6^ Community participation that is inclusive of underserved groups and is tailored to context is a fundamental principle of equitable primary health care as well as a way of optimizing interventions to improve health.

## Participatory approaches

In this paper we examine the concepts of participation and co-production in health care with a focus on health services and communities working together to achieve health goals. Here we define communities as groups of people who share common interests, concerns or identities in settings that are defined by geography, culture, administrative boundaries or geopolitical region or that are identified with joint activities, such as work or recreation.^7^^,^^8^

Participatory approaches and the characteristics of participation have been defined in different ways.^9^ Some authors^10^ distinguish between organic participation such as community-organized actions, contrasting this with induced participation that is externally stimulated. Members of the community may be involved in the latter type to a greater or lesser extent, with participation ranging from outreach and consultation at one end of the spectrum of participation to collaboration and shared leadership at the other end.^11^ Countries or programmes may move along the spectrum as they gain experience or according to their objectives.

In this paper we discuss externally-stimulated community participation that falls at the collaboration and shared leadership end of the participation spectrum. This is not to say the burden of resolving health issues is placed on communities. To be transformative, participatory approaches in health require power-sharing with health-service users. This is likely to mean new relationships, including a new culture in health-care institutions that supports participation.^9^^,^^12^

Participation does not usually operate as a linear intervention to improve health; rather participatory approaches form a set of complex processes and interactions.^10^^,^^11^^,^^13^ An approach that is based on systems theory is useful to understand participation processes, whereby interdependencies between different parts of a system are explicitly recognized and nonlinear effects are expected to occur and are taken into account.^14^

Issues of power and control should be considered, both to understand systems better and to ensure that participatory interventions do not unintentionally reinforce potentially harmful social structures.^2^^,^^8^ This means looking at who is engaged, why and in what way. For example, including only men as participants in a programme might reinforce pre-existing gender inequities. Failing to seek out underserved groups may further entrench inequities.

## Areas for action

To achieve social transformation, participatory approaches in health need to work alongside each other at different levels. We identify three areas for action: (i) individual and community capabilities to participate; (ii) people-centred health services; and (iii) social accountability. These areas align with existing frameworks such as the capabilities approach^15^ and health promotion charters^16^ that highlight the need to attend to factors outside the traditional realm of health services, including the role of different participants in the production of health.

The three areas are interrelated and should be addressed in parallel. For instance, without improved capabilities for participation on all sides, it will be a challenge to introduce community participation in quality improvement efforts in support of a more people-centred health service. A supportive policy environment that identifies social accountability mechanisms will legitimize and support participatory processes at all levels.

### Improving capabilities

For individuals to develop as agents of change and for participatory processes to work well, individuals and groups need the capabilities to achieve the health goals they value.^15^

Facilitated participatory learning and action cycles with women’s groups have been identified as an effective way to build individual and group capabilities, to identify and prioritize problems and to develop a plan for implementing locally feasible strategies to address these (Box 1).^17^ Freire’s concept of critical consciousness (a deepened awareness of the social, political and economic situation, including health, that leads one to understand that one can intervene and that these realities can be transformed) has also been emphasized in participatory learning.^18^ Others have proposed that all humans need to feel competent, autonomous and related to others.^19^^,^^20^ A positive social environment can yield better psychological, developmental and behavioural outcomes by helping meet these three key needs, that is, by supporting people in their activities and giving them a sense of volition and choice.^19^^,^^20^ When supported to develop their own skills, individuals may voluntarily support others, for example by sharing breastfeeding techniques or setting up support groups, thereby introducing a layer of sustainability independent of the original intervention.

Box 1 **The four phases of participatory learning and action cycles**

Facilitated participatory learning and action cycles with women’s groups involve a four-phase participatory process with a trained facilitator, in which women’s groups collectively decide on priority actions, and try to organize activities accordingly. The cycle is structured as follows: (i) identify and prioritize problems that may occur during pregnancy, childbirth and after birth; (ii) plan activities; (iii) implement strategies to address the priority problems; (iv) assess the activities and plan changes as needed.^17^

Supportive environments, therefore, are also key determinants of good health and healthy practices (Box 2).^22^ Unsupportive environments in society may, for example, deter young people from obtaining condoms or women from breastfeeding in public. Participatory interventions encourage dialogue and can help identify socially- and culturally-acceptable solutions.^23^ For instance, strategies to promote men’s involvement during pregnancy, childbirth and after birth are recommended to improve care practices for women and newborns. However, these interventions should be designed in dialogue with women to avoid undermining women’s choices, autonomy and decision-making.^24^

Box 2 **Example of a participatory approach to strengthen community support for pregnant women**

In Andhra Pradesh state, India, a participatory intervention attempted to increase demand for quality care through meetings to raise awareness and increase community support for pregnant women; involving families (particularly husbands) in pregnancy-related care; and bi-monthly home visits by a community organizer who helped families access care and create birth preparedness plans. Local elected leaders also held regular meetings to review performance of public health providers and facilities. Afterwards, women reported changes in support received from family members during pregnancy and childbirth and decreased workload during their pregnancy.^21^

### People-centred services

A key goal in global health is creating people-centred health services, that is, services orientated around the needs and preferences of users rather than around diseases.^25^ Achieving this requires participatory approaches. Participation of service users in planning, governance and quality improvement processes, as well as community partnerships with services, can help to make health services and health professionals more responsive to the needs of their clients and the wider community.^11^^,^^24^^–^^26^ The World Health Organization recommends community participation in quality improvement processes for maternity care services and in programme planning and implementation to improve maternal and newborn health.^24^ Others have emphasized the importance of community empowerment for improving care services for women after an abortion.^27^

Participation by members of underserved groups may stimulate services towards more equitable provision of care.^25^^,^^28^ Attempts to address the needs of excluded groups have included engaging community members as mediators or employing health staff from the relevant culture, for example, to develop culturally-appropriate maternity care services. Another approach is strengthening efforts to build stronger relationships and dialogue between communities, institutions and service providers about the care required.

Social transformation requires the people who are in control to share their power.^18^ This principle has been taken up in the National Health Service in England, which aims to create a culture of shared decision-making, aspiring to create equal partnerships between clinicians, patients and carers and with patients involved in co-design, co-commissioning and, overall, co-production of health care.^29^ Converting the aspirations into reality is not easy, although there have been successes (Box 3).^12^

Box 3 **Example of patient participation and co-production in health services quality improvement**

In the National Health Service in England, long-term ethnographic study has shown how projects involving patients, clinicians and researchers working together in non-hierarchical teams have helped empower patient participants to collaborate with clinicians to devise health-care improvements.^30^ For example, a patient-held record called My Medication Passport was developed by and for patients, particularly those using multiple services. It is a patient-completed aide memoire containing information about the patient’s clinical history and medication. Patients can carry this to appointments to facilitate communication with clinicians, as well as between clinical teams.^31^ Patient participants have also played a key role in linking up clinical and non-clinical services.^14^

Participatory health interventions require an interactive approach by health-care providers that changes the usual patient–provider dynamic. The skills of health-care providers may need to be developed to help them collaborate with service users or community members^32^^,^^33^ and to move from solving problems for patients, to solving problems with patients. Health facility leadership that supports this type of collaboration is also essential for people-centred care.^25^ A better work environment and greater job satisfaction can improve health-care workers’ sense of autonomy and their motivation to engage in respectful care and quality improvement processes.^34^^,^^35^ If health-care workers appear reluctant to engage in dialogue with patients, the overall work environment needs to be examined to help understand why this is and to address any institutional barriers to health-care workers’ autonomy and motivation.

### Social accountability

Accountability is central for progress in women’s, children’s and adolescents’ health.^1^ Citizens’ voices are important to build equitable health systems and to provide quality health services, particularly in settings with poor governance.^36^

The World Bank identifies four factors vital for any social accountability programme: (i) the opportunities for information exchange, dialogue and negotiation between citizens and the state; (ii) the willingness and ability of citizens and civil society to seek government accountability; (iii) the willingness and ability of service providers and policy-makers to support constructive engagement with citizens; and (iv) the broader environment that enables increased civic engagement (such as the policy, legal and regulatory environment; the type of political system, the values and norms of society).^37^

Members of the community need to recognize their entitlement to health but also understand the constraints of health systems. This enables them to play an integral role in planning, implementing, monitoring and evaluating policies and services, and identifying workable solutions. Common strategies for participation in accountability processes include community representation in health facility management committees,^26^ village health committees, community taskforces or citizens’ hearings,^38^ as part of community-monitoring processes (Box 4).^11^ These processes are dynamic: skills are needed to achieve dialogue and to build the trust required for the different participants to plan and work together.^24^ Implementation efforts should raise awareness among individuals and communities (for example, by providing information about health services or promoting awareness of entitlements) and address aspects of the social context that might affect participation (such as fear of speaking out). Health-service managers can take first steps to gain confidence in participatory processes, such as setting up complaints or comments boxes for patients to use, or publishing health-services statistics to inform and prompt discussion between health, development and community stakeholders. Once they gain experience they can move on to more sophisticated engagement processes.^39^^,^^40^

Box 4 **Example of improving service delivery with participatory monitoring**

In a randomized field experiment of community-based monitoring of primary health-care providers in the public health system in Uganda, community perceptions of health facilities were summarized into report cards. A community meeting was held to present the reports to the community and share information about health rights. Community members and health staff developed action plans including details of how the community would monitor agreed actions. Service uptake and quality of services improved. After one year, for instance, 36% of treatment facilities had a suggestion box, compared with none in the control facilities. The treatment facilities posted more information on free services and patient rights and obligations, were kept in better condition, had higher uptake of vaccinations and better indicators of service use than the control facilities. The study also reported a 33% reduction in under-five mortality.^39^

## Challenges

Participation is frequently emphasized in global, regional and national health policies, yet we lack examples of large-scale, transformative action in practice. Some challenges to implementation of participatory approaches are outlined below.

Not all approaches described as community or participatory successfully achieve participation or transformation. Our experience suggests this may be because these concepts are often too broadly applied. There may be little open, ongoing dialogue, or no attempt to achieve the collaborative styles of working that are essential for co-production in health care. Selecting and training community health workers, for example, is often classified as a community participation approach. While many community health workers deliver information or services outside clinics or provide a link between communities and health services, they do not necessarily represent the views of the community nor do their tasks necessarily require them to consult with the community. Similarly, nongovernmental organizations (NGOs) are sometimes seen to reflect the voice of the community, despite not having consulted with members of communities or been nominated as their representatives.^41^

Community members may become more committed, engaged and motivated if they see positive results from participatory activities.^12^ Conversely, communities may become disengaged from the process if there is no obvious positive effect of engagement or, for instance, if workings of committees are not transparent.^41^ A study in Malawi found that attempts by NGOs to foster participation can create expectations that cannot be maintained and can potentially undermine other ways for the community to participate.^42^

Hierarchies within health care may make adopting and integrating participatory approaches especially difficult to achieve. For example, if there is no open dialogue about quality improvement within the health service, engaging the users of the service in the discussion will also be difficult. A participatory, respectful management philosophy in the system as a whole provides a foundation for transforming relations with communities.^12^^,^^35^ It is not enough simply to tell others to use these approaches with community members. For example, if central- or regional-level managers do not communicate with district-level staff, or if different service providers (such as doctors, midwives) do not feel respected by one another and do not work as a team, they are less likely to have the empathy or motivation required for positive, transformative encounters with the community. Health-care workers may also resist the processes of accountability for fear of reprimand or punishment.

Good intentions about participatory approaches may not always be accompanied by the support needed for implementation. The Government of India has promoted a system for women to register complaints with health facility managers or through patient welfare committees. Reports, however, suggest that the procedures may not be clear and that women are not always aware they can make a complaint against a doctor or nurse or they fear reprisals if they do so.^43^

How to scale up participatory approaches is not always clear from the literature, despite the case studies and success stories that exist at district level. The level of ambition demonstrated by the initiative in Odisha state of India remains rare (Box 5).

Box 5 **Example of scaling up participatory interventions**

Facilitated participatory learning and action cycles with women’s groups (see Box 1) to address maternal and newborn health in poor rural communities were shown to improve newborn health outcomes.^44^ A meta-analysis of studies from Bangladesh, India, Malawi and Nepal showed newborn deaths decreased by one third in settings where groups with at least 30% of pregnant women came together to address issues around the health of the mother and the baby.^17^

Scale-up of women’s groups has been undertaken in Odisha state, India. Local experience of greater equity after an intervention involving women’s groups, combined with a state-level emphasis on evidence-based policy, prompted a new community dialogue intervention called Shakti Varta. Launched in 2013, Shakti Varta was intended to cover a population of 20 million people across 15 districts (half of the state) who have many health and nutrition challenges.^45^ Challenges common to scale-up efforts have been encountered; for instance how to ensure fidelity to the important elements of the intervention across the project districts, and how to ensure high-quality training and implementation.

Finally, the existing literature reveals little agreement on how to evaluate the impact of participatory approaches. The existence of participatory activities can be demonstrated but it is often difficult to link them to health outcomes. There are several reasons for this: (i) because of the complexities of linking social change directly to health outcomes; (ii) because participatory activities often take place within a package of interventions and so the effects of participation cannot be separated out; and (iii) because health outcomes are so strongly influenced by the performance of the health sector and other social determinants. Furthermore, health improvements and uptake of services are only part of the story; measurement of participatory approaches should also account for any resulting social change, such as changes in equitable use of services, changes in social and gender dynamics, and issues relating to sustainability.^10^^,^^11^

## Discussion

Achieving transformative action towards improving the health of women, children and adolescents will depend on deploying locally appropriate participatory approaches at community, health service and policy level across the three action areas described in this paper. These contributions will influence whether proven biomedical health interventions reach the intended populations and whether the global goals for sustainable development are met.

The challenge is that to be transformative, power must be shared with health service users. To do this entails building new relationships and fostering a new culture in health-care institutions that is supportive of participatory approaches.^9^^,^^12^ Participatory approaches need to be embedded throughout the health system: internally (within health-care teams and between levels of the health system), as well as externally (between services and communities). 

Participatory approaches are particularly important in decentralized health systems where local leadership is promoted, because there is more opportunity for bottom-up partnership between communities and health services, including in decision-making processes and community accountability mechanisms.^46^

Interventions can fail because of poor design and implementation. Programme managers and practitioners need to pay attention to detail in implementing participatory approaches, just as an immunization campaign needs to pay attention to the cold-chain supply or to staff training. For example, to build awareness and mobilize the community around a specific issue, attention should be paid to how facilitators are selected and trained, who is participating in the meetings and why, whether training manuals for participants are appropriate to their experience and education, whether meetings are held at convenient times and places, whether there is adequate coverage and frequency of those meetings and how information is shared among peers.

Although participation may be regarded as desirable in itself, participatory approaches are neither widely practised nor well documented. There is a growing evidence base supporting the use of participatory approaches to improve health, but concerted effort is needed to develop better and more relevant measures of participatory interventions and to gain agreement on what is to be measured and how. Factors that are difficult to measure, such as social change, may often be ignored. To understand the mechanisms and dimensions of participation and transformative action, it will be important to measure such factors more adequately.

More guidance on specific community-oriented interventions is required to help inform country and donor investments. We need to understand what works, how different approaches can work in different contexts and what factors need to be taken into account for future scaling up and sustainability of interventions.^47^

All three objectives of the global strategy – survive, thrive and transform – will benefit from participatory approaches in the areas we have described. Indeed, to some extent, progress towards the sustainable development goals could be measured by the presence of mechanisms to enable participatory approaches and co-production in health care to develop in practice.

We know much already about the power of participation. In a sense it is no longer a technical issue, but one of civil rights and political will. For transformative action on women’s, children’s and adolescents’ health, participatory approaches are essential, at all levels: district, national, regional and global. Without these, we face the risk of stalled progress and persisting inequities in health.
